# Quantitative assessment of the choroidal vasculature in myopic macular degeneration with optical coherence tomographic angiography

**DOI:** 10.3389/fopht.2023.1202445

**Published:** 2023-06-14

**Authors:** Yee Shan Dan, Kai Xiong Cheong, Shen Yi Lim, Qiu Ying Wong, Rachel S. Chong, Chee Wai Wong, Quan V. Hoang

**Affiliations:** ^1^ Singapore Eye Research Institute, Singapore National Eye Centre, Singapore, Singapore; ^2^ Ophthalmology & Visual Sciences Academic Clinical Program (Eye ACP), Duke-NUS Medical School, Singapore, Singapore; ^3^ Department of Ophthalmology, Yong Loo Lin School of Medicine, National University of Singapore, Singapore, Singapore; ^4^ Department of Ophthalmology, Edward S. Harkness Eye Institute, Columbia University Vagelos College of Physicians and Surgeons, New York City, NY, United States

**Keywords:** myopic macular degeneration, optical coherence tomographic angiography, choroidal thickness, choroidal vessel density, choroidal branch area, choroidal vessel width, medium vessel, large vessel

## Abstract

**Background:**

To assess and compare choroidal morphometric vascular parameters, using optical coherence tomographic angiography (OCTA), in highly myopic adults with and without myopic macular degeneration (MMD).

**Methods:**

This is a clinic-based observational study of 148 eyes with axial length (AL) ≥25mm, enrolled from the high myopia clinic of the Singapore National Eye Centre. MMD was graded from fundus photographs. Swept source OCT (SS-OCT) and OCTA were performed and assessed for choroidal layer thickness (CT) and choroidal vasculature (choroidal vessel density (CVD), choroidal branch area (CBA) and mean choroidal vessel width (MCVW)) in the different choroidal layers (overall choroidal layer (CL), medium-vessel choroidal layer (MVCL), large-vessel choroidal layer (LVCL)).

**Results:**

CT_CL_ (r=-0.58, p<0.001), CT_MVCL_ (r=-0.22, p=0.04), MCVW_CL_ (r=-0.58, p<0.001), and CVD_CL_ (r=-0.19, p=0.02) were negatively correlated with AL, while CBA_CL_ (r=0.61, p<0.001) was positively correlated. Compared to eyes with no MMD, eyes with MMD2 had lower CT_CL_ (120.37±47.18µm vs 218.33±92.70µm, p<0.001), CT_MVCL_ (70.57±15.28µm vs 85.32±23.71µm, p=0.04), CT_LVCL_ (101.65±25.36µm vs 154.55±68.41µm, p=0.001) and greater CVD_CL_ (71.10±3.97% vs 66.97±3.63%, p<0.001), CVD_MVCL_ (66.96±2.35% vs 65.06±2.69%, p=0.002), CVD_LVCL_ (68.36±2.56% vs 66.58±2.88%, p=0.012), MCVW_MVCL_ (6.14±0.34µm vs 5.90±0.35µm, p=0.007), and CBA_CL_ (12.69±1.38% vs 11.34±1.18%, p<0.001). After adjusting for age, thicker CT_CL_ (odds ratio (OR) 0.98, 95% confidence interval (CI) 0.97-0.99, p<0.001), CT_MVCL_ (OR 0.97 (0.94-0.99), p=0.002) and CT_LVCL_ (OR 0.97 (0.96-0.98, p<0.001) were significantly associated with lower odds of MMD2, while increased CVD_CL_ (OR 1.37 (1.20-1.55), p<0.001), CVD_MVCL_ (OR 1.39 (1.12-1.73), p=0.003), CVD_LVCL_ (OR 1.31 (1.07-1.60), p=0.009), CBA_CL_ (OR 2.19 (1.55-3.08), p<0.001) and MCVW_MVCL_ (OR 6.97 (1.59-30.51), p=0.01) was significantly associated with higher odds of MMD2.

**Conclusion:**

Decrease in choroidal vessel width, density and thickness, and an increase in vascular branching were observed in eyes with long AL. A thinner and denser choroid with greater branching area and vessel width, which may all be signs of hypoxia, were associated with greater odds of MMD2.

## Introduction

Myopic macular degeneration (MMD) is a major cause of visual impairment and blindness globally. As high myopia and age are the major risk factors for MMD, an epidemic of MMD is expected in the near future, especially in East Asian countries where the prevalence of high myopia is 7% to 22% (1–3). Although our knowledge of the pathologic changes in high myopia has advanced since the past decade, a lot is still unknown about the pathogenesis of MMD. One of the unmet needs in the clinical management of high myopia lies in predicting those who are at the greatest risk of MMD progression. This may now be possible with advances in imaging technology that have enabled the *in vivo* study of the retina/choroid/sclera complex, some or all of which are likely to be the primary protagonists in the pathogenesis of MMD (1, 4–6).

The advent of optical coherence tomographic angiography (OCTA) enabled imaging of the choroidal vasculature non-invasively, making it possible to assess the choroid beyond the single dimension of choroidal thickness. Our group had previously utilized swept source optical coherence tomography (SS-OCT) to identify decreased choroidal thickness (CT) as a key feature in MMD, and demonstrated that CT was more strongly associated with MMD than scleral thickness (7). This finding suggests that analysis of the choroidal layer may provide further clues to the pathogenesis of MMD. However, it was still unclear if choroidal thinning in MMD stems from atrophy of the choroidal vasculature or stroma.

OCTA remains limited in the visualization of the deeper vascular layers of the choroid, mostly because of scattering by the pigment in the RPE and by vessels in the choriocapillaris (CC), and consequent signal attenuation (8). Moreover, the CC produces strong OCTA projection artifacts in the underlying choroid. Because of the signal attenuation, choroidal vessels are typically visualized as silhouettes with complete loss of signal at greater depths. Consistent visualization of the medium and larger-sized vessels of Sattler’s and Haller’s layers may be better appreciated in eyes with areas of overlying RPE loss or depigmentation with absence of the CC, permitting greater penetration of light into the choroid.

Currently, these limitations have been partially overcome by using ultrahigh-speed SS-OCTA instruments (9). Recently, the choroid has been imaged with a novel research device with a Fourier-domain mode-locked laser system operating with a 1.7- MHz sweep rate (1.7 million A-scans/s), at a 1065-nm center wavelength. In a recent study, this device was used to obtain OCTA images with a phase-variance method and these images were qualitatively compared with those obtained with an SD-OCTA instrument operating with a 68 kHz imaging rate and an 840-nm center wavelength (10). In this comparison, the authors noted an improved visualization of the medium and larger-sized choroidal vessels in the images obtained using the SS-OCTA with increased imaging speed. Notably, the visualization of the choroid with this device was demonstrated to be even better than that obtained with ICGA, since the latter imaging modality does not resolve the meshwork of capillary vessels and has limited capability to visualize the medium-sized vessels. However, given these limitations in the visualization of the deeper choroidal vascular layers using existing commercial instruments, most OCTA studies thus far have focused on the CC, providing the greatest amount of information for this thin dense layer of capillaries, both in healthy and affected eyes.

Similarly in one of our follow-up study, we demonstrated qualitatively the presence of choriocapillaris signal voids on OCTA in eyes with MMD (11). The degree of choriocapillaris signal voids was more extensive than that indicated by the severity of MMD, suggesting that OCTA evaluation of the CC may detect pathological changes before the onset of clinically apparent atrophy. We postulated that choriocapillaris changes suggest that the pathogenesis of MMD may be related to the Bruch’s membrane, which is essentially the basement membrane of the choriocapillaris. However, we did not evaluate changes in the deeper layers of the choroid, namely the Sattler’s and Haller’s layers, and whether vascular changes in these deeper layers could be the cause of alterations in the choriocapillaris. To address this, our group developed a novel segmentation technique correlating OCTA images and the anatomical OCT structure of the choroid that allowed the detailed vascular analysis of Haller’s and Sattler’s layers in high myopes (11).

In this study, we aim to evaluate changes in the medium and large choroidal vessel layers of high myopes with MMD using the aforementioned technique.

## Methods

### Study population

This was a clinic-based observational clinical study in which patients with high myopia aged 21-70 years, with manifest spherical equivalent (SE) of ≤ -5.0 dioptres (D) or axial length (AL) of ≥ 25mm in either eye, were recruited from the High Myopia clinic of the Singapore National Eye Centre from January 2017 to December 2017. Both eyes of a patient may be included for analysis if the eye has a SE of ≤ -5.0 D or AL of ≥ 25mm. Patients with any coexisting or previous ocular disease in either eye, including corneal opacities, uveitis, dense cataracts, vitreous haemorrhage, diabetic retinopathy or diabetic macular edema, central serous chorioretinopathy, previous retinal laser photocoagulation or photodynamic therapy, previous and coexisting retinal detachment, retinal dystrophies, macular scarring from any cause other than myopic maculopathy, retinopathy due to any cause other than myopia, previous retinal vein or artery occlusion, and ocular ischemic syndrome were excluded from this study. This study was approved by the Centralized Institutional Review Board (CIRB) of SingHealth, Singapore (protocol number R1364/50/2016) and conducted in accordance with the Declaration of Helsinki. All participants provided signed informed consent for their participation.

### Clinical examination

All patients underwent comprehensive ophthalmic evaluation including best corrected visual acuity (BCVA), measured with the logarithm of the minimal angle of resolution (logMAR) chart (Lighthouse International, New York, New York, USA) at 4 meters, slit lamp biomicroscopy examination, and manifest refraction performed by certified study optometrists. Intraocular pressure was measured with Goldmann applanation tonometry. Spherical equivalent (SE) was calculated as the sum of the spherical power and half of the cylindrical power. The IOL Master (Carl Zeiss Meditec AG, Jena, Germany) was used for ocular biometry including AL, anterior chamber depth (ACD) and keratometry measurements.

### Imaging protocol and image grading

Fundus photography centered at the fovea was performed with a 45-degree digital retinal camera after pupillary dilation with tropicamide 1% and phenylephrine 2.5%, using Canon CR-DGi with Canon EOS 10D SLR backing (Canon Inc, Tokyo, Japan). The optical coherence tomography (OCT) images were obtained using a swept source OCT device (Topcon DRI OCT Triton, Topcon, Tokyo, Japan). This instrument uses a light source with a central wavelength of 1050 nm and an acquisition speed of 100,000 A scans per second. 12mm raster and radial scans centered on the macula and optic nerve were obtained. Fundus photographs and OCT images were graded by 2 retinal specialists (CWW, QVH) for retinal and optic nerve lesions.

### Grading of MMD

Two retinal specialists (CWW, QVH), masked to participant characteristics performed grading of the fundus photographs using the International Meta-Analysis for Pathologic Myopia classification.

### Segmentation of choroidal layers

**Figure 1:**
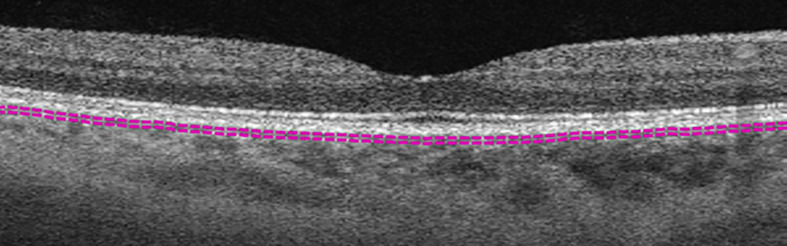
Example of choriocapillaris layer marked by default segmentation lines (purple dashed lines).

**Figure 2:**
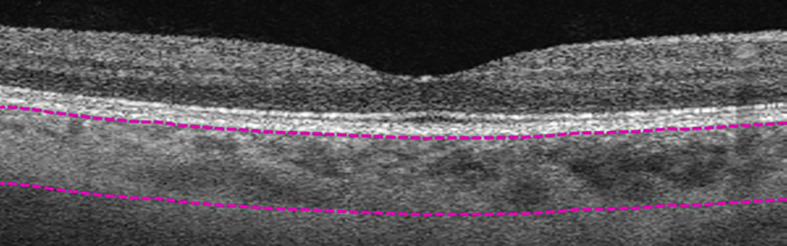
Manual segmentation of overall choroidal layer (CL). Top segmentation line (top purple dashed line) sits at the innermost border of the choroidal layer, while the bottom segmentation line (lower purple dashed line) was shifted to the outermost large choroidal vessel, marking the CL as the layer between the purple segmentation lines.

**Figure 3:**
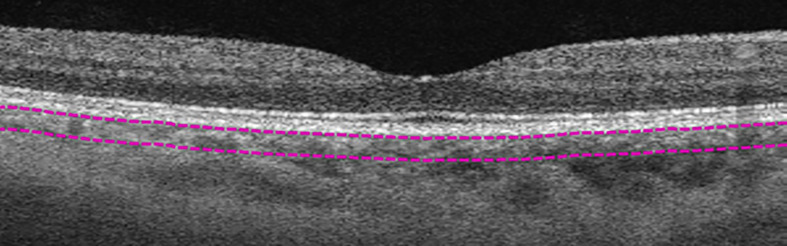
Manual segmentation of medium-vessel choroidal layer (MVCL). Top segmentation line (top purple dashed line) sits at the innermost border of the choroidal layer, while the bottom segmentation line (lower purple dashed line) was shifted to the innermost large choroidal vessel, marking the MVCL between the purple dashed segmentation lines.

**Figure 4:**
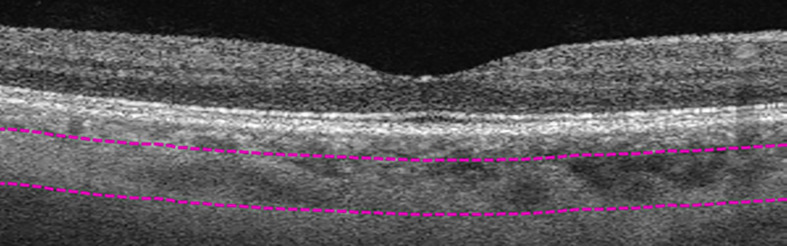
Manual segmentation of large-vessel choroidal layer (LVCL). Top segmentation line (top purple dashed line) sits at the innermost large choroidal vessel, while the bottom segmentation line (lower purple dashed line) was shifted to the outermost large choroidal vessel, marking the LVCL between the purple dashed segmentation lines.

3mm×3mm images from the Plex Elite (PLEX Elite, Carl Zeiss Meditec, Dublin, CA) were used for the analysis of choroidal layers. The highest-quality foveal cut of the OCT B-scans was chosen by two experienced fellowship-trained retinal specialists (CWW and QVH) for choroidal thickness grading. Only one highest-quality B-scan, closest to the foveal center was used for segmentation. The choroid was manually segmented by two masked observers, with excellent reliability (ICC = 0.987) into overall choroidal layer (CL), medium-vessel choroidal layer (MVCL), and large-vessel choroidal layer (LVCL) according to the technique described by Devarajan et al., 2019 (11). Using the PLEX Elite software, the choriocapillaris layer presented with default segmentation lines close to the Bruch’s membrane (**Figure 1**). CL was segmented by adjusting the top segmentation line to the Bruch’s membrane – for images where the default segmentation lines are at the wrong position – and moving the bottom segmentation line to the suprachoroid, which is indicated by the lowest visible large vessel (**Figure 2**). MVCL was segmented next by moving the bottom segmentation line above the highest point of the hyporeflective large vessels, leaving MVCL with a hyperreflective appearance comprising of the small and medium vessels of the Sattler’s layer (**Figure 3**). LVCL segmentation was performed by subtracting MVCL from CL, giving this layer the appearance of only hyporeflective large vessels of the Haller’s layer (**Figure 4**). The thickness of each layer was calculated by subtracting the value of the top segmentation line from the bottom segmentation line (in µm). Eyes with overall choroidal thickness less than 100 µm were excluded as it was not possible to have reliable segmentation of the choroid into the MVCL and LVCL sublayers.

**Figure 5:**
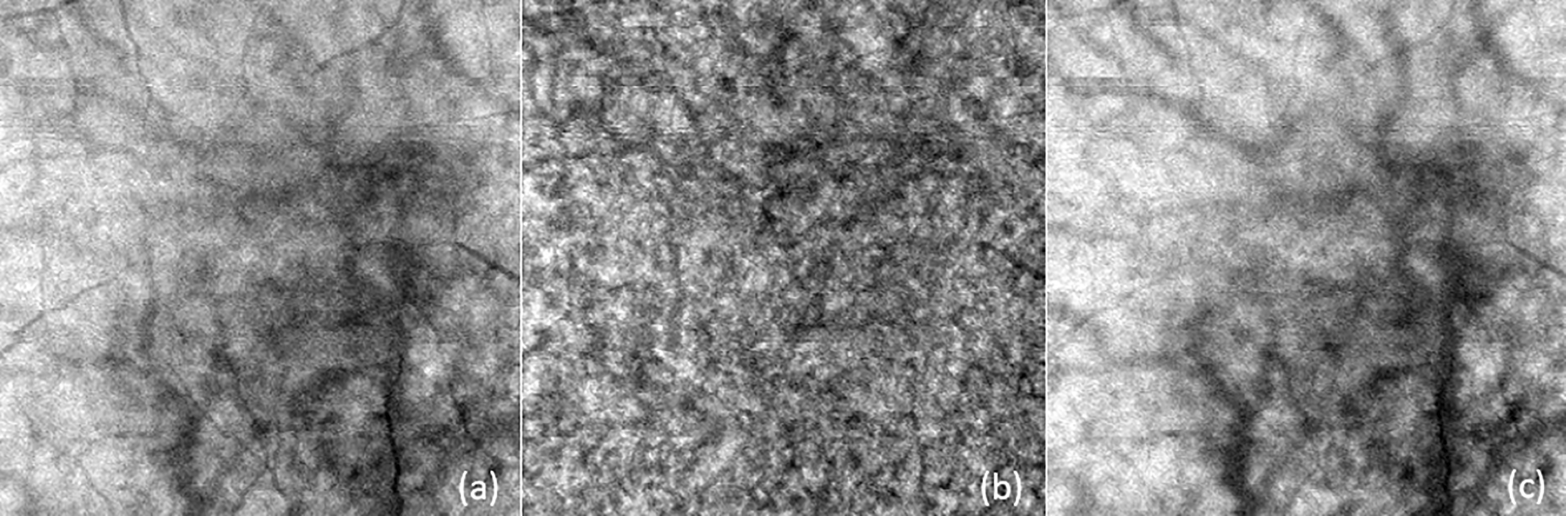
Examples of enface images for **(A)** overall choroidal layer (CL), **(B)** medium vessel choroidal layer (MVCL), and **(C)** large vessel choroidal layer (LVCL). These images were subsequently processed with Matlab for choroidal vasculature parameters: choroidal vessel density (CVD), choroidal branch area (CBA), and mean choroidal vessel width (MCVW).

Enface images obtained from segmenting the different layers were compiled and processed using Matlab as described by Devarajan et al., 2019 (11) for choroidal vasculature parameters: choroidal vessel density (CVD, %), choroidal branch area (CBA, %), and mean choroidal vessel width (MCVW, µm) see **Figure 5**.

A total of 12 choroidal vascular parameters were obtained: overall choroidal layer thickness (CT_CL_), medium-vessel choroidal layer thickness (CT_MVCL_), large-vessel choroidal layer thickness (CT_LVCL_), choroidal vessel density – overall choroidal layer (CVD_CL_), choroidal vessel density – medium-vessel choroidal layer (CVD_MVCL_), choroidal vessel density – large-vessel choroidal layer (CVD_LVCL_), choroidal branch area – overall choroidal layer (CBA_CL_), choroidal branch area – medium-vessel choroidal layer (CBA_MVCL_), choroidal branch area – large-vessel choroidal layer (CBA_LVCL_), mean choroidal vessel width – overall choroidal layer (MCVW_CL_), mean choroidal vessel width – medium-vessel choroidal layer (MCVW_MVCL_), and mean choroidal vessel width – large-vessel choroidal layer (MCVW_LVCL_).

### Statistical analysis

All data were expressed as mean ± standard deviation or proportions as appropriate. Means were compared with the two-sample Kolmogorov-Smirnov test. Binary logistic regression analysis was performed to identify independent factors associated with poorer visual acuity, adjusted for age. Each individual’s left and right eye was accounted for as fixed effects. Each choroidal morphometric vascular parameter was corrected for magnification error calculated using Littman’s method and the Bennett formula, F = 3.48 × 0.01306 × (AL – 1.82), where F represents the magnification factor and AL represents axial length. A p-value of <0.05 was considered to be statistically significant. All statistical analyses were performed using Stata/MP 16.1 (College Station, TX 77845, USA).

## Results

In the original cohort, there were very few eyes that were META-PM category 3 or 4 (five and two, respectively). In addition, we were also unable to segment the choroid into sublayers for such eyes with very thin choroids. We therefore excluded the META-PM category 3 and 4 eyes. A total of 148 eyes were included and were split into two groups for analysis, with 74 eyes in the MMD2 group and 74 eyes in the no MMD group.

Patients with MMD2 were significantly older (56.4 ± 12.0 vs. 51.2 ± 12.2 years, p = 0.009) and had longer AL (29.0 ± 1.6 vs. 27.4 ± 1.4 mm, p < 0.001) than those without see **Table 1**.

**Table 1:** Patient characteristics.

	Overall (n=148)	MMD2 (n=74)	No MMD (n=74)	P-value
Age, years	53.8±12.3	56.4±12.0	51.2±12.2	0.009*
Gender (male), %	39.2%	35.1%	43.2%	0.97
AL, mm	28.2±1.7	29.0±1.6	27.4±1.4	< 0.001*
logMAR BCVA	0.22±0.16	0.24±0.15	0.21±0.17	0.78

Asterisk and bold font denote P < 0.05. AL, axial length; BCVA, best-corrected visual acuity; MMD, myopic macular degeneration.

Age and 12 choroidal vascular parameters were analysed for any significant correlation with AL, BCVA and CT. Longer AL was associated with thinner CT_CL_ (r = -0.58, p < 0.001), thinner CT_MVCL_ (r = -0.22, p = 0.04), lower CVD_CL_ (r = -0.19, p = 0.02), reduced MCVW_CL_ (r = -0.58, p < 0.001), and greater CBA_CL_ (r = 0.61, p < 0.001). Poorer BCVA was associated with thinner CT_CL_ (r = -0.21, p = 0.01). Thinner CT_CL_ was also associated with older age (r = -0.38, p < 0.001), and greater CBA_CL_ (r = -0.60, p < 0.001) with an equal increase in CBA_MVCL_ and CBA_LVCL_ (r = -0.22, p = 0.04 for both). Thinner CT_CL_ was also associated with thinner CT_MVCL_ (r = 0.79, p < 0.001) and CT_LVCL_ (r = 0.98, p < 0.001), decreased CVD_CL_ (r = 0.38, p < 0.001) with significant decrease in CVD_LVCL_ (r = 0.41, p < 0.001), and reduced MCVW_CL_ (r = 0.61, p < 0.001) also with significant decrease in MCVW_LVCL_ (r = 0.27, p = 0.01) but the changes for CVD_MVCL_ and MCVW_MVCL_ were not considered as significant (p ≥ 0.05) (**Table 2**).

**Table 2:** Correlation of age and choroidal vascular parameters with AL, BCVA and CT.

	AL		BCVA		CT_CL_	
	r	P-value	r	P-value	r	P-value
Age	0.09	0.27	0.12	0.16	-0.38	< 0.001*
CT_CL_	-0.58	< 0.001*	-0.21	0.01*	–	–
CT_MVCL_	-0.22	0.04*	-0.13	0.23	0.79	< 0.001*
CT_LVCL_	-0.19	0.81	-0.21	0.05	0.98	< 0.001*
CVD_CL_	-0.19	0.02*	-0.15	0.07	0.38	< 0.001*
CVD_MVCL_	-0.19	0.08	0.13	0.22	0.02	0.86
CVD_LVCL_	-0.08	0.46	-0.06	0.60	0.41	<0.001*
CBA_CL_	0.61	< 0.001*	0.01	0.88	-0.60	< 0.001*
CBA_MVCL_	0.19	0.07	-0.18	0.10	-0.22	0.04*
CBA_LVCL_	0.18	0.09	-0.04	0.72	-0.22	0.04*
MCVW_CL_	-0.58	< 0.001*	-0.03	0.68	0.61	< 0.001*
MCVW_MVCL_	-0.19	0.07	0.19	0.08	0.21	0.06
MCVW_LVCL_	-0.18	0.09	0.02	0.82	0.27	0.01*

Each choroidal morphometric vascular parameter was corrected for magnification error. Asterisk and bold font denote P < 0.05. AL, axial length; BCVA, best-corrected visual acuity; CBA, choroidal branch area; CL, overall choroidal layer; CT, choroidal layer thickness; CVD, choroidal vessel density; LVCL, large-vessel choroidal layer; MCVW, mean choroidal vessel width; MVCL, medium-vessel choroidal layer. Magnification error was calculated using Littman’s method and the Bennett formula *F* = 3.48 × (*AL*-1.82), where F represents the magnification factor and AL represents axial length.

Comparison of the choroidal vascular parameters between eyes with MMD2 and with no MMD revealed that eyes with MMD2 have thinner CT_CL_ (120.37±47.18µm vs 218.33±92.70µm, p<0.001), along with thinner CT_MVCL_ (70.57±15.28µm vs 85.32±23.71µm, p=0.04) and CT_LVCL_ (101.65±25.36µm vs 154.55±68.41µm, p=0.001). But had greater CVD_CL_ (71.10±3.97% vs 66.97±3.63%, p<0.001), CVD_MVCL_ (66.96±2.35% vs 65.06±2.69%, p=0.002), CVD_LVCL_ (68.36±2.56% vs 66.58±2.88%, p=0.012), MCVW_MVCL_ (6.14±0.34µm vs 5.90±0.35µm, p=0.007), and greater CBA_CL_ (12.69±1.38% vs 11.34±1.18%, p<0.001) in eyes with MMD2 (**Table 3**).

**Table 3:** Comparison of choroidal vascular parameters between eyes with MMD2 versus no MMD.

	Overall (n=148)	MMD2 (n=74)	No MMD (n=74)	P-value*
CT_CL_	169.35±88.25	120.37±47.18	218.33±92.70	< 0.001*
**CT_MVCL_	80.84±22.48	70.57±15.28	85.32±23.71	0.04*
**CT_LVCL_	138.50±63.50	101.65±25.36	154.55±68.41	0.001*
CVD_CL_	69.03±4.32	71.10±3.97	66.97±3.63	<0.001*
CVD_MVCL_	65.62±2.73	66.96±2.35	65.06±2.69	0.002*
CVD_LVCL_	67.11±2.89	68.36±2.56	66.58±2.88	0.012*
CBA_CL_	12.02±1.45	12.69±1.38	11.34±1.18	< 0.001*
CBA_MVCL_	12.74±0.89	12.88±0.93	12.68±0.88	0.15
CBA_LVCL_	10.52±0.97	10.81±0.94	10.40±0.97	0.13
MCVW_CL_	6.91±0.44	6.94±0.42	6.89±0.46	0.29
MCVW_MVCL_	5.97±0.36	6.14±0.34	5.90±0.35	0.007*
MCVW_LVCL_	7.42±0.62	7.50±0.68	7.39±0.60	0.93

Means of the MMD2 group and no MMD group were compared using the two-sample Kolmogorov-Smirnov test. Each choroidal morphometric vascular parameter was corrected for magnification error. Asterisk and bold font denote P < 0.05. **N=62 for the No-MMD group and N=27 for the MMD2 group for MVCL and LVCL comparisons. CBA = choroidal branch area. CL, overall choroidal layer; CT, choroidal layer thickness; CVD, choroidal vessel density; LVCL, large-vessel choroidal layer; MCVW, mean choroidal vessel width; MMD, myopic macular degeneration; MVCL, medium-vessel choroidal layer. Magnification error was calculated using Littman’s method and the Bennett formula *F* = 3.48 × 0.01306 × (*AL*-1.82), where F represents the magnification factor and AL represents axial length.

Binary logistic regression, prior to adjusting for age (unadjusted odds ratio), revealed thicker CT_CL_ (odds ratio (OR) 0.98, 95% confidence interval (CI) 0.97-0.98, p < 0.001), thicker CT_MVCL_ (OR 0.96, 95% CI 0.94-0.99, p = 0.001) and thicker CT_LVCL_ (OR 0.97, 95% CI 0.96-0.98, p < 0.001) were associated with lower odds of MMD2. Presence of MMD2 was associated with greater odds of increased CVD_CL_ (OR 1.35, 95% CI 1.20-1.53, p < 0.001), increased CVD_MVCL_ (OR 1.34, 95% CI 1.10-1.63, p = 0.004), increased CVD_LVCL_ (OR 1.26, 95% CI 1.05-1.51, p = 0.01), increased CBA_CL_ (OR 2.25, 95% CI 1.60-3.17, p < 0.001) and increased MCVW_MVCL_ (OR 6.79, 95% CI 1.53-30.22, p = 0.01). After adjusting for age (adjusted odds ratio), thicker CT_CL_ (OR 0.98, 95% CI 0.97-0.99, p < 0.001), thicker CT_MVCL_ (OR 0.97, 95% CI 0.94-0.99, p = 0.002) and thicker CT_LVCL_ (OR 0.97, 95% CI 0.96-0.98, p < 0.001) remained associated with lower odds of MMD2. Presence of MMD2 remained significantly associated with greater odds of increased CVD_CL_ (OR 1.37, 95% CI 1.20-1.55, p < 0.001), increased CVD_MVCL_ (OR 1.39, 95% CI 1.12-1.73, p = 0.003), increased CVD_LVCL_ (OR 1.31, 95% CI 1.07-1.60, p = 0.009), increased CBA_CL_ (OR 2.19, 95% CI 1.55-3.08, p < 0.001) and increased MCVW_MVCL_ (OR 6.97, 95% CI 1.59-30.51, p = 0.01) (**Table 4**).

**Table 4:** Association of choroidal vascular parameters with presence of MMD2.

	Unadjusted Odds ratio	95% CI	P-value	Adjusted odds ratio	95% CI	P-value
CT_CL_	0.98	0.97-0.98	< 0.001*	0.98	0.97-0.99	< 0.001*
CT_MVCL_	0.96	0.94-0.99	0.001*	0.97	0.94-0.99	0.002*
CT_LVCL_	0.97	0.96-0.98	< 0.001*	0.97	0.96-0.98	<0.001*
CVD_CL_	1.35	1.20-1.53	< 0.001*	1.37	1.20-1.55	<0.001*
CVD_MVCL_	1.34	1.10-1.63	0.004*	1.39	1.12-1.73	0.003*
CVD_LVCL_	1.26	1.05-1.51	0.01*	1.31	1.07-1.60	0.009*
CBA_CL_	2.25	1.60-3.17	< 0.001*	2.19	1.55-3.08	<0.001*
CBA_MVCL_	1.29	0.75-2.24	0.36	1.35	0.78-2.33	0.29
CBA_LVCL_	1.58	0.94-2.67	0.09	1.54	0.91-2.61	0.11
MCVW_CL_	1.36	0.64-2.86	0.42	1.65	0.73-3.76	0.23
MCVW_MVCL_	6.79	1.53-30.2	0.01*	6.97	1.59-30.51	0.01*
MCVW_LVCL_	1.32	0.62-2.81	0.47	1.45	0.67-3.17	0.35

“Adjusted odds ratio” was adjusted for age. Each choroidal morphometric vascular parameter was corrected for magnification error. Each individual’s left and right eye was accounted for as fixed effects. Asterisk and bold font denote P < 0.05. CBA, choroidal branch area; CL, overall choroidal layer; CT, choroidal layer thickness; CVD, choroidal vessel density; LVCL, large-vessel choroidal layer; MCVW, mean choroidal vessel width; MVCL, medium-vessel choroidal layer; Magnification error was calculated using Littman’s method and the Bennett formula, *F* = 3.48 × 0.01306 × (*AL*-1.82), where F represents the magnification factor and AL represents axial length.

## Discussion

Consistent with previous studies (12–14), our study found a strong correlation between AL and CT, where the longer the AL, the thinner the CT_CL_ (r = -0.58, p < 0.001). Besides CT_CL_, CVD_CL_ (r = -0.19, p = 0.02) and MCVW_CL_ (r = -0.58, p < 0.001) were also noted to decrease with longer AL, while CBA_CL_ increases with longer AL (r = 0.61, p < 0.001). None of the parameters for the middle- and large-vessel choroidal layers, except for CT_MVCL_ (r = -0.22, p = 0.04) were significantly associated with changes in AL. Other investigators had noted similar findings for the negative correlation for CT_CL_, CVD_CL_ and MCVW_CL_ with AL, and positive correlation for CBA_CL_ with AL (15–18). Taken together, these findings suggest that the increase in AL could have exerted a stretching force on the choroidal vessels, thereby decreasing choroidal vessel width. With the decrease in vessel width, there would be a decrease in blood flow which would affect the choroidal thermoregulation (19). Thus, this could have led to the increase in CBA, which could be an increase in choroidal vascular branching or elongation or both, in an attempt by the eye to regulate the retinal/choroidal blood supply, similar to the mechanism where choroidal neovascularisation (CNV) occurs. Interestingly, blood flow in choroidal vessels has been reported to be lower in highly myopic versus non-highly myopic eyes, with a possible negative correlation between axial length and choroidal blood flow (20). This supports the idea that the thinning of MCVW could have led to a noticeable decrease in blood flow and vessel branching increase could be a compensatory mechanism to overcome the limitation in blood flow. Further investigation is necessary to verify if the molecular mechanisms of choroidal vessel changes in elongated, pathological eyes have any similarities to CNV.

Thinner CT_CL_ was also associated with worse BCVA (r = -0.21, p = 0.01), older age (r = -0.38, p < 0.001), and thinner CT_MVCL_ and CT_LVCL_ (r = 0.79 and r = 0.98, p < 0.001 for both), mirroring what several other groups had observed (12–14, 21, 22). CVD_CL_ was also noted to be positively correlated with CT_CL_ (r = 0.38, p < 0.001), with the changes in CVD_LVCL_ being the main driver (r = 0.41, p < 0.001). Wang et al. (2020) similarly noted that CVD_LVCL_ was larger in patients with central serous chorioretinopathy (CSC) as compared to controls (62.3 ± 6.6% vs. 54.1 ± 8.0%, p = 0.02) (23). Although these eyes were affected by a different disease, it seems that regardless of the type of disease, CVD_LVCL_ appears to play a more impactful role than CVD_MVCL_ for changes in the overall choroidal layer.

Our understanding of the changes in CVD could be further broken down through understanding the changes for CBA and MVCW, with MCVW_CL_ (r = 0.61, p < 0.001) and MCVW_LVCL_ (r = 0.27, p = 0.01) noted to have a significant positive correlation with changes in CT as well. Together with the results for CVD, the large-vessel choroidal layer appears to be a more active player in driving changes for the overall choroidal layer than the medium-vessel choroidal layer as the correlation for CVD_MVCL_ was not significant (p = 0.86) and MCVW_MVCL_ was marginally significant (p = 0.06). For CBA_CL_ (r = -0.60, p < 0.001), it was negatively correlated with CT_CL_, with equal contributions of both MVCL and LVCL (r = -0.22, p = 0.04 for both) affecting the increase in the choroidal branch area. From the changes in CBA and CVD, it seemed that changes for MCVW were probably larger than that of the choroidal branching (which could either be an increase in vessel branching or increased length of vessels) to result in overall changes in CVD to be similarly negatively correlated with CT changes.

Several choroidal vasculature parameters that we studied (CT_CL_, CT_MVCL_, CT_LVCL_, CVD_CL_, CVD_MVCL_, CVD_LVCL_, CBA_CL_, and MCVW_MVCL_) were significantly altered in the presence of MMD2 (**Table 3**). Compared to eyes with no MMD, MMD2 eyes showed thinner CT_CL_ (120.37µm vs 218.33µm, p < 0.001), which was consistent for both MVCL (70.57µm vs 85.32µm, p = 0.04) and LVCL (101.65µm vs 154.55µm, p=0.001). This finding is similar to other studies, in which Wong et al. noted that CT was highly correlated to MMD (7), and Zhou et al. similarly noted that CT is thinner in more myopic eyes (24). Meanwhile, Zhao et al. also found that MVCL was only significantly associated with a higher prevalence of some, and not all, macular diseases (14).

Interestingly, it was noted by Yang and Koh that impediment of ocular blood was possibly associated with axial length increase, especially so in high myopes (20). In the present study, we find three choroidal vascular parameters that may reflect worsened hypoxia (**Table 3**). For example, compared to eyes with no MMD, choroidal vessel density was consistently higher in MMD2 eyes for CVD_CL_ (71.10 vs. 66.97%, p < 0.001) as well as for CVD_MVCL_ and CVD_LVCL_ (p = 0.002 and p = 0.012). Moreover, MCVW_MVCL_ was greater in MMD2 eyes versus eyes with no MMD (6.14 vs. 5.90 µm, p = 0.007). Additionally, the choroidal branching area (CBA_CL_) was significantly greater in eyes with MMD2 (12.69 vs. 11.34%, p < 0.001). As discussed further below, hypoxia, specifically in the choriocapillaris layer could readily result in a compensatory response in medium- as well as the large-vessel layers in attempts improve perfusion with increased vessel density, increased vessel width and increased branching area.

Thicker CT_CL_, with significant contributions from both MVCL and LVCL, were significantly associated with lower odds of MMD2, after adjusting for age (OR 0.98, 95% CI 0.97-0.99, p < 0.001; OR 0.97, 95% CI 0.94-0.99, p = 0.002; OR 0.97, 95% CI 0.96-0.98, p < 0.001, respectively). As mentioned above, a compensatory response to hypoxia in the choriocapillaris likely explains our finding that presence of MMD2 increases the odds of higher CVD (of CL, MVCL and LVCL), CBA_CL_ and MCVW_MVCL_. Another possible explanation for the increase in CVD could be the increased risk of myopic complications in MMD eyes. MMD eyes are known to have a higher risk of developing posterior staphylomas and myopic CNV, with both features being associated with pronounced choroidal thinning (19, 25, 26). Limitations in discerning the individual choroidal layers within staphyloma eyes leave open the possibility of an increased CVD within the MVCL and LVCL as some sort of defense mechanism which the eye could have taken to prevent staphyloma formation/progression. As the choriocapillaris is not readily resolvable in standard OCTA imaging, it could very well be the changes in the MVCL and LVCL mask changes occurring in the much thinner choriocapillaris, which would contribute relatively less to the overall change in the CL. Also, as it is currently unknown which choroidal layer myopic neovascularization originates from (19), we cannot eliminate the possibility of mCNV originating from the MVCL or LVCL versus the choroicapillaris. With overall thinning of the choroid in MMD eyes, the increase in CVD_MVCL_ and CVD_LVCL_ could be a defensive mechanism where the choroid tries to regulate choroidal blood flow to prevent hypoxia. Similarly, an increase in vessel width (MCVW_MVCL_) could be a similar defense mechanism. Moreover, past studies have suggested that a response to hypoxia could be increased vessel branching (27). Increased branching would result in increased CVD. Hypoxia may very well explain why the presence of MMD increases the odds of greater CBA, CVD and MCVW.

Our study is not without its limitations, some of which are discussed above. In the original cohort, there were very few eyes that were META-PM category 3 or 4 (five and two, respectively). In addition, we were also unable to segment the choroid into the MVCL and LVCL sublayers for such eyes which would have extremely thin choroids. We therefore excluded the META-PM category 3 and 4 eyes. Hence, this study was not designed to assess the effect of MMD severity, and we could only assess the effect of its presence. Furthermore, our study has a cross-sectional design and this precludes the inference of a temporal or sequential relationship. In this regard, we cannot tell which choroidal layer the main changes in axial elongation occur in. Moreover, we did not account for chronic conditions such as diabetes, kidney disease and hypertension. These conditions are associated with changes in the choroidal vasculature (20), which could lead to a different interpretation of results if they were taken into account. Also, questions remain on whether choroidal vessels appeared thinner mainly due to the mechanical stretching forces or if they were congenitally thinner in eyes with a higher risk of myopia (28). Histological studies had revealed that the choroid thins with age and increased refractive error, but the cause of choroidal thinning remains poorly understood. Comparing choroidal thickness in similar AL could provide a better idea of how different the choroidal vessels are. Staphyloma, mCNV and other MMD-related features were not investigated in this study as well, which could have provided us with a better idea of why CVD_MVCL_ increases with an increase in odds of MMD.

In conclusion, longer eyes were observed to have decreased choroidal vessel width, reduced choroidal vessel density, thinner choroidal layer, and increased vascular branching. The latter could be a compensatory mechanism to maintain choroidal blood supply in elongated eyes. A thinner choroid, with almost equal MVCL and LVCL thinning, and denser MVCL, which may be signs of hypoxia, were also associated with greater odds of MMD.

## Data availability statement

The raw data supporting the conclusions of this article will be made available by the authors, without undue reservation.

## Ethics statement

The studies involving human participants were reviewed and approved by Centralized Institutional Review Board (CIRB) of SingHealth, Singapore. The patients/participants provided their written informed consent to participate in this study.

## Author contributions

YD and KC drafted the manuscript. SL performed all statistical analysis and created all tables. QW helped acquire the data. RC, CW and QH conceived the project, acquired the data and directed the writing and analysis. All authors proofread the manuscript. All authors contributed to the article and approved the submitted version.
